# Comparison of Th‐2 Cytokines and Associated Gene Expression in Peripheral Blood of Resistant and Susceptible Garole Sheep Infected With *Haemonchus contortus*

**DOI:** 10.1155/japr/3052032

**Published:** 2026-04-15

**Authors:** Ananta Hembram, Supradip Das, Soumitra Pandit, Surajit Baidya, Abhijit Nandi, Subhas Chandra Mandal, Shyam Sundar Kesh, Shamik Polley, Amlan Patra, Ayan Mukherjee, Ruma Jas

**Affiliations:** ^1^ Department of Veterinary Parasitology, West Bengal University of Animal and Fishery Sciences, Kolkata, West Bengal, India, wbuafscl.ac.in; ^2^ Department of Veterinary Clinical Complex, West Bengal University of Animal and Fishery Sciences, Kolkata, West Bengal, India, wbuafscl.ac.in; ^3^ Department of Veterinary Biochemistry, West Bengal University of Animal and Fishery Sciences, Kolkata, West Bengal, India, wbuafscl.ac.in; ^4^ American Institute for Goat Research, Langston University, Langston, Oklahoma, USA, langston.edu; ^5^ Department of Animal Biotechnology, West Bengal University of Animal and Fishery Sciences, Kolkata, West Bengal, India, wbuafscl.ac.in

**Keywords:** Garole sheep, *Haemonchus contortus*, immune response, resistance, Th-2 cytokines

## Abstract

Level of Th‐2 cytokines (IL‐4, IL‐5, and IL‐13) in serum and relative expression of associated cytokine genes (*IL-4*, *IL-5*, and *IL-13*) in peripheral blood along with other parameters of host resistance were compared between the resistant and susceptible Garole sheep infected with *Haemonchus contortus*. Resistant (*n* = 12) and susceptible Garole sheep (*n* = 12) were selected based on faecal egg count (FEC) and packed cell volume (PCV) and were divided into three equal groups: healthy control, infected resistant, and infected susceptible. Sheep of infected groups were orally challenged with infective larvae of *H*. *contortus* and then FEC, body weight, haemoglobin (Hb), PCV, peripheral eosinophil counts (PEC), and serum Th‐2 cytokines concentration and associated gene (*IL-4*, *IL-5,* and *IL-13*) expressions were measured. Partial sequences of all the three cytokine genes were also analysed. Significantly (*p* < 0.05) lower FEC was observed in resistant sheep with no eggs in faeces from 31 to 35 DPI while the susceptible sheep continued to pass *Haemonchus* eggs through faeces till end of the study. Body weight, Hb, PCV, and PEC of resistant sheep were higher (*p* < 0.05) compared with susceptible sheep. Concentrations of Th‐2 cytokines (IL‐4, IL‐5, and IL‐13) as well as expression all the genes (*IL-4*, *IL-5*, and *IL-*13) were found to be greater (*p* < 0.05) in resistant sheep on different days post infection. No single nucleotide variation was observed in either of the three genes that could be suggested to be responsible host resistance. In resistant sheep, increased eosinophilia, Th‐2 cytokine concentration, and relative expression Th‐2 cytokine genes in peripheral blood were observed synchronously with that of the elimination of parasites indicating the role of those components in resistance against *H*. *contortus* and this needs to be explored by examining local reaction in the abomasum of infected host.

## 1. Introduction

Gastrointestinal nematode (GIN) infection poses significant economic decrement in the livestock industry, particularly in small ruminant rearing systems. Among the nematodes affecting the small ruminants, *Haemonchus contortus* is the most pathogenic one, leading to severe anaemia and sometimes death in acute infection [1]. Chronic infection by this species precipitates a reduction in growth rate, milk production, and reproduction rate [2, 3]. Favorable epizootiological determinants for faster growth and transmission of this parasite, the nomadic nature of the herds, poor socioeconomic status, and traditional managemental practices have been major contributing factors for the high prevalence of this parasitic infection across the world.

Control of this parasitic infection always remains a challenging task and has been a subject of interest for researchers. Strategic application of control measures against this parasitic infection mainly relies on chemotherapy. However, chemotherapeutic control measures, despite their popularity, suffer from the inherent limitations of the development of drug‐resistant strains of parasites and residual effects in livestock products. Available literature exhibits that *H. contortus* has been resistant to almost all classes of anthelmintics [4, 5].

An array of research works alternative to chemotherapy were carried out all over the world and also in progress of late. Amongst the strategies alternative to chemotherapy, use of animals genetically resistant to GIN in breeding program is one of the most promising methods which was the prime interest of the earlier workers and in the recent past as well. The strategies are being developed to envisage the actual reason of host resistance using immunological and molecular biological tools. The researchers are consistently working in this direction and they are pondering on the nuanced area of it to unravel the markers usable for selection of resistant animals. Host immune response is crucial in protection against GIN and thereby plays a significant role in host resistance [6, 7]. Identification of specific immunological events/factors responsible for host resistance is a prerequisite for selection of resistant animals. Now it is evidently a fact that both cell mediated and humoral immune response attribute to the development of host resistance against GIN infection. Humoral immune response against GIN infection is synchronized by Th‐2 lymphocytes which releases cytokines like IL‐4, IL‐5, IL‐9, IL‐10, and IL‐13 in response to infection [8–10]. Th‐2 cytokines help in accumulation and activation of eosinophils, mast cell and other effector cells [11]. The activated eosinophils release again different Th‐2 cytokines that regulate B lymphocytes for differentiation of immunoglobulin (Ig) particularly IgE against GIN antigens. Host immune response is regulated by the type of cytokines released during the course of GIN infection [12]. Th‐2 lymphocytes coordinated the immune response by secreting different types of cytokines, recruiting eosinophils and mucosal mast cells and also production of IgA, IgE, and IgG and all these actively take part in reducing the rate of survival and establishment of GIN infection [13] in the host.

Garole sheep are found in the Sundarban delta of West Bengal especially in the coastline saline zone of the south and north 24 Parganas and parts of East Midnapore district [14]. Garole sheep are mainly reared by the small and marginal farmers following a semi‐intensive system without any provision of good nutrition and routine deworming. Garole sheep are a highly prolific breed and are also famous for low‐fat good quality mutton, and this breed is also being used in cross‐breeding programs with other sheep breeds to increase their growth rate [15]. The population of Garole sheep experienced a notable decrease in its native region, reducing from 0.27 million in 2003 to 0.16 million by 2013 [16] indicating an urgent need for the conservation of this breed. Garole sheep are highly adaptable to hot and humid, saline, and flood‐prone conditions of the Sundarban delta. Garole sheep are known to be resistant to foot‐rot, a common disease of sheep. Garole sheep also exhibited resistance against GIN infections [17, 18] including *H*. *contortus* [19, 20], the most pathogenic GIN of sheep.

Different sets of experiments [17–23] were carried out earlier in our laboratory in this direction to envisage how the interaction of various host immunological factors provides the resistance in Garole sheep against *H. contortus*. The results of different parameters such as FEC, determination of serum IgG and IgA levels along with the relative expression level of IFN‐*γ* gene (Interferon gamma) strongly suggest the development of resistance against *H. contortus* in Garole sheep. The role of Th‐2 cytokines as well as Th‐2 cytokine genes for mounting host resistance has not been studied earlier in Garole sheep although Th‐2 type of immune response is believed to influence host resistance against GIN. As the role of Th‐1 cytokine (IFN‐*γ*) and its respective gene (*IFN-γ* gene) on host resistance has been unraveled in Garole sheep by comparing the concentration of cytokine IFN‐*γ* and relative expression of *IFN-γ* gene in peripheral circulation [20]; now it is a dire need to explore the role of Th‐2 cytokines and their respective genes in Garole sheep as well. Taking into account those observations, the present study was designed to evaluate the impact of Th‐2 immune response on host resistance by comparing serum concentration of Th‐2 cytokines and relative expression of associated genes in peripheral blood along with other phenotypic markers of resistance between resistant and susceptible Garole sheep infected with *H*. *contortus*.

## 2. Materials and Methods

### 2.1. Selection of Resistant and Susceptible Garole Sheep

A total of 60 Garole sheep of either sex within the age group of 3–6 months were selected in the sheep unit of the West Bengal University of Animal and Fishery Sciences, Mohanpur, Nadia district, West Bengal, India. They were identified by their metallic ear‐tags throughout the entire period of study. In the respective unit, the sheep were maintained by a semi‐intensive system of rearing where they were allowed for grazing during the daytime and stall‐fed at night. They were dewormed at 3‐month intervals in the unit. However, during the study period of 1 year, a regular deworming schedule was not followed for the animals used in the present experiment, and they were provided with anthelmintic as and when required. After the completion of the 1‐year field study, all the Garole sheep showing gastrointestinal (GI) parasitic infection by qualitative fecal examination were treated with anthelmintics.

Perrectal fecal samples were collected from all the identified sheep at monthly interval for a duration of 1 year. The faecal samples were brought to the laboratory using a top cork plastic specimen collecting container. The samples were examined by standard sedimentation and floatation technique [24]. Quantitative examination of fecal sample for estimation of FEC of individual sheep was done by Modified McMaster technique [24]. After FEC estimation of all the selected sheep for a duration of 1 year, 20 sheep (*n* = 20) showing persistently low FEC were identified as resistant to GIN infection and the rest 40 sheep (*n* = 40) exhibiting variable FEC were identified as susceptible. Sheep showing low FEC ranging from 0 to 350 with a mean FEC value ≤ 150 during the 1 year field study were considered as resistant. Although sheep showing variable FEC (0–2500) with a mean FEC value ≥ 500 in 1 year were identified as susceptible. The mean FEC value of resistant sheep (*n* = 20) varied from 79.17 (±22.58) to 133.33 (±36.58) and that of susceptible sheep (*n* = 40) ranged from 716.67 (±127.23) to 820.83 (±198.62). After the end of field study, blood was collected from all the 20 resistant sheep and 20 susceptible sheep which showed always higher FEC (≥ 500) compared with other susceptible sheep in 1 year of the field study. The parameter, packed cell volume (PCV) was kept in this study as an indicator of resilience against blood sucking *Haemonchus* infection, and PCV of all the 40 sheep (resistant sheep = 20 and susceptible sheep = 20) was estimated once by Wintrobe′s haematocrit method [25]. As mentioned earlier, resistant and susceptible Garole sheep were so classified based on analyzing FEC data, the phenotypic indicator of resistance, and similarly, PCV data was considered as a phenotypic marker of resilience. Finally, 12 female Garole sheep persistently exhibiting low FEC (mean EPG ≤ 150) and higher PCV (PCV > 33 ranging from 33.4 to 36) were established as resistant sheep. A comparable methodological approach was employed to determine susceptible animals. A total of 12 female Garole sheep showing variable FEC (mean EPG ≥ 700) and lower PCV (PCV < 30; ranging from 26.8 to 29.8) were considered as susceptible sheep. After the final selection is over, all the resistant Garole sheep (n = 12; avg weight = 11.258 kg ± 0.23) and susceptible Garole sheep (*n* = 12; avg weight = 10.767 kg ± 0.138) were brought to the university experimental animal house to pursue further experimental work.

### 2.2. Maintenance and Grouping of Experimental Sheep

All the selected animals were treated with subcutaneous administration of Doramectin at 0.2 mg/kg body weight and maintained under strict intensive system in the experimental animal house of the university. The animals were provided with healthy ration consisting adequate green fodder (tree leaves), hay (chopped straw), and recommended quantity of concentrate feed regularly with provision of clean drinking water. A keen observation on the animal′s health was kept consistently throughout the entire period of study. They were maintained in this status for about 3 months in the animal house before giving artificial infection of *H*. *contortus* larvae. The sheep were divided into three groups, that is, healthy control (*n* = 8; four resistant and four susceptible sheep), infected resistant (*n* = 8), and infected susceptible (*n* = 8). The animals of distinct groups were maintained in segregated housing units.

### 2.3. Experimental Infection, Fecal Egg Count, and Body Weight

Adult female *H. contortus* collected from the abomasum of slaughtered sheep were triturated in phosphate buffer saline (PBS) using a pestle and mortar for collection of eggs. The eggs were subject to culture for harvesting third stage infective larvae (L_3_) following fecal culture technique [24]. For procurement of sufficient number of infective larvae of *H. contortus* required for experimental infection, one nondescript donor sheep was reared intensively in the animal house for 3 months in GI parasite free status. Fecal sample of donor sheep was collected as per the method mentioned earlier and examined by standard sedimentation and floatation technique [24] for two occasions at 3 days interval. Once it was confirmed that the donor animal is free from any GI parasitic infection as reflected by fecal examination, the animal was infected orally with *H*. *contortus* larvae (L_3_) at a dose of 500 L_3_/kg body weight following an overnight period of food deprivation [20] and subsequently regular ration and drinking water was provided to the animal as per standard schedule. After the donor sheep became patent, culture of feces of that animal was started. Feces were collected in feces collecting bags and the same was cultured following a standard technique [24] to harvest infective larvae of *H*. *contortus*. The infective larvae were harvested after 7 days of coproculture. They were made free of fecal debris and concentrated by conventional Baermann technique [26]. The larvae so collected were finally preserved in distilled water at 4°C for downstream application in experimental infection of the Garole sheep. Prior to artificial infection routine fecal examination of all the experimental sheep was done on three alternate days following standard method [24] to ensure the GI parasite free status of the animals. After recording the body weight, the sheep of both the resistant and susceptible groups were orally infected with *H. contortus* larvae as described above for donor sheep.

After the onset of patency in all the infected resistant and susceptible sheep, fecal sample examination was started and corresponding FEC of all the infected sheep was recorded employing modified McMaster technique [24] on every alternate day from 21 days post infection (DPI) up to 41 DPI for 11 occasions. Fecal samples of all the control sheep were also examined by standard technique [24]. Body weights were simultaneously recorded for all the experimental sheep at 10 days intervals from 0 to 40 DPI.

### 2.4. Blood and Serum Samples

Approximately 5 mL of blood was aseptically collected by venipuncture of the jugular vein of each of the experimental sheep using a well‐branded sterile disposable plastic syringe from 0 DPI to 40 DPI at 10‐day intervals. Out of this 5 mL blood, 3 mL was kept in an EDTA vial for the purpose of hematological studies, RNA extraction and cDNA synthesis from each of the samples. Remaining 2 mL of blood was kept undisturbed in the syringe itself for a period of 4 h for serum separation. Then the separated serum was transferred to vials and duly preserved at −20°C for further use in the Enzyme Linked Immunosorbent Assay (ELISA).

### 2.5. Hematological Studies

Hemoglobin (gm/dl) and PCV% of all the experimental sheep were carried out following Drabkin′s method [27] and microhaematocrit method [25], respectively. Total leukocyte counts and differential counts were estimated by the method of Jain [28]. Then the eosinophil percentage of total leukocyte was recorded. Absolute peripheral eosinophil count was determined by multiplying the eosinophil percent with total leukocytes [29].

### 2.6. Serum IL‐4, IL‐5, and IL‐13 Concentration

Commercial ELISA kits from Bethyl Laboratories, United States were employed to determine the serum concentration of IL‐4, IL‐5, and IL‐13 in all the experimental Garole sheep from 0 DPI to 40 DPI at 10‐day intervals. Sandwich ELISA was performed as per the kit′s instruction utilizing precoated micro‐ELISA plates with anti‐ovine IL‐4, IL‐5, and IL‐13 antibodies. The optical density (OD) values of each well were recorded at 450 nm in ELISA Reader (ThermoFisher Scientific, United States). For determination of concentration of the aforesaid cytokines, a standard curve was generated by plotting the OD values of the standard solution of known concentration. The mean OD values at 450 nm were obtained and concentration of different cytokines in serum were determined by comparing the OD values of the test samples with the OD values of standard solution utilizing the standard curve prepared as mentioned already. The units for concentrations of serum IL‐4 and IL‐5 were pg/ml and the value of IL‐13 was expressed in ng/ml.

### 2.7. Expression Profile of IL‐4, IL‐5, and IL‐13 Genes

To record the level of mRNA expression of *IL-4*, *IL-5*, and *IL-13* genes, synthesis of cDNA is prerequisite. In our study, cDNA was synthesized from whole RNA extracted from peripheral blood mononuclear cells (PBMC) following a standard protocol of reverse transcription reaction. A gradient centrifugation [30] was systematically carried out for isolation of PBMC from the whole blood. Total RNA was extracted using PureZol (BioRad, United States) following standard method [31] and then the RNA samples were processed to remove any DNA contamination using DNAse (Merck, Sigma Aldrich, United States). It is a crucial step to ensure the extracted RNA is of high quality and free from genomic DNA contamination. Both the concentration and quality of isolated RNA samples were duly determined by employing a Nanodrop spectrophotometer (Eppendorf, Germany). The quality of the RNA samples was analysed by running RNA samples in denaturing agarose gel (1%) electrophoresis ( S1).

Reverse transcription reaction was carried out to synthesize cDNA from the extracted whole RNA samples placed in 20 *μ*l reaction mixtures using iScript Reverse Transcriptase cDNA synthesis kit (BioRad, United States) as per the manufacturer′s instruction. The freshly prepared cDNA was preserved at −20°C for future use in Reverse Transcription Quantitative Polymerase Chain Reaction (RT‐qPCR). One ovine housekeeping gene, that is, Glyceraldehyde‐3‐Phosphate Dehydrogenase (*GAPDH*) was taken as reference gene for RT‐qPCR. This housekeeping gene was amplified to validate the synthesised cDNA by conventional PCR using specific primers (Table 1). Amplification of *IL-4*, *IL-5*, and *IL-13* genes from the synthesized cDNA was done by performing conventional PCR using specific primers (Table 1). Determination of annealing temperature is important prerequisite for setting up the PCR running. The annealing temperatures were standardized for all three target genes including housekeeping gene by performing conventional gradient PCR following standard protocol. The gradient PCR revealed annealing temperatures for *GAPDH*, *IL-4*, *IL-5,* and *IL-13* genes as 60°C, 57°C, 60°C, and 55°C, respectively. The temperatures were well documented for utilization of the data during RT‐q PCR.

**Table 1:** Primer sequences, amplicon size and annealing temperatures standardized for ovine *GAPDH*, *IL-4*, *IL-5*, and *IL-13* genes.

Sl. No.	Gene	Primer sequence	Amplicon size	Type of PCR	Tm (°C)
1	*GAPDH*	Forward: GGGTCATCATCTCTGCACCT	176 bp	RT‐qPCR	60°C
		Reverse: GGGTCATCATCTCTGCACCT			
2	*IL-4*	Forward: CGCTGAACATCCTCACATCG	190 bp	RT‐qPCR	57°C
		Reverse: AGGCTGCTGAGATTCCTGTC			
3	*IL-5*	Forward: GAATCAAACTGCACAAGGGGAT	137 bp	RT‐qPCR	60°C
		Reverse: CTTGCAGGTAGTCGAGGAATTG			
4	*IL-13*	Forward: AGAACCAGAAGGTGCCGCT	51 bp	RT‐qPCR	55°C
		Reverse: GGTTGAGGCTCCACACCATG			
5	*IL-4*	Forward: GTTGGCAGCACTTCAGTTGG	741 bp	Conventional PCR	57°C
		Reverse: GGTGAAGCAGGGGGAAATCA			
6	*IL-5*	Forward: TGTGAGGTCAGTCAGTATCGT	618 bp	Conventional PCR	57°C
		Reverse: TCTCCTCCACACTTCCTCTG			
7	*IL-13*	Forward: GGAGCTGACCCTAATAATGG	524 bp	Conventional PCR	54°C
		Reverse: CTTCTGTGTTGTTCTAGGCT			

Abbreviations: *GAPDH*, ovine *GAPDH* gene; *IL-4*, ovine cytokine *IL-4* gene; *IL-5*, ovine cytokine *IL-5* gene; *IL-13*, ovine cytokine *IL-15* gene; and Tm, melting temperature (annealing temperature).

The RT‐qPCR is a powerful and sensitive technique used to measure gene expression level and it was carried out employing a commercial qPCR kit (Sso Fast Eva Green, BioRad, United States) and RT‐PCR machine (CFX touch, BioRad, United States) operational with BioRad CFX manager software. The reaction mixture for RT‐qPCR (10 *μ*l) was prepared using SsoFastEvaGreenSupermix (5 *μ*l; BioRad, United States), forward primer (0.25 *μ*l), reverse primer (0.25 *μ*l), cDNA template (0.5 *μ*l), and nuclease‐free water (4 *μ*l). After repeated running of RT‐qPCR, the reaction protocol was standardized. The data being unfolded during standardization were for Step I (enzyme activation at 95°C for 30 s), Step II (denaturation at 95°C for 5 s), Step III (annealing/extension for 10 s), Step II to Step III repeated for 39 times, and finally Step IV (Melt curve at 65°C–95°C for 5 s in 0.5°C increment).

As mentioned earlier, the relative expression of *IL-4*, *IL-5*, and *IL-13* genes was determined by performing RT‐qPCR of all those genes along with *GAPDH* and semiquantitative gene expression analysis. The normalization was done using test CT values and CT values of *GAPDH* (endogenous housekeeping control gene). The fold changes in the expression levels of test cytokine genes were derived with the expression values relative to the control sheep at different DPI as per the method proposed by Pfaffl [32]. The RT‐qPCR products of *GAPDH*, *IL-4*, *IL-5*, and *IL-13* genes were checked by performing conventional gel electrophoresis in 1% agarose gel. The bands were visualized under UV light and analysed on a Gel Documentation System (GelDoc, BioRad, United States). The RT‐qPCR products of *GAPDH, IL-4*, *IL-5,* and *IL-13* genes were subject to gel electrophoresis revealing 176 bp, 190 bp, 137 bp, and 51 bp amplicon sizes, respectively (Figures S2, S3, S4, and S5, respectively).

### 2.8. Amplification and Sequencing of Th‐2 Cytokine Genes From Genomic DNA

Genomic DNA was extracted from the peripheral blood of all the experimental sheep (*n* = 24) consisting of 12 resistant sheep and 12 susceptible sheep selected earlier for experimental *H*. *contortus* infection. Genomic DNA was retrieved utilizing a commercially procured Blood DNA extraction kit (Qiagen, Netherlands) as per manufacturer′s instruction. The quality and concentration of isolated DNA were checked in a Nanodrop spectrophotometer (Eppendorf, Germany). The appropriate primers were designed as per standard primer‐designing software using the available gene sequences in NCBI data base and bioinformatics tools (Table 1). The Th‐2 cytokine genes *viz*., *IL-4*, *IL-5*, and *IL-13* were amplified by conventional PCR in a 25 *μ*l reaction mix using the specific primers in a thermal cycler (BioRad, United States). For amplification of the partial sequence of Th‐2 cytokine genes, the cycling conditions standardized as initial denaturation (at 95°C for 5 min), 35 cycles of denaturation (at 95°C for 1 min), and annealing at 57°C (for *IL-4* and *IL-5* genes) and 54°C (for *IL-13* gene) for 1 min, and also extension at 72°C for 1 min and final extension at 72°C for 10 min. The PCR products of *IL-4*, *IL-5*, and *IL-13* genes were subject to Gel Electrophoresis (1% agarose) in horizontal electrophoresis apparatus (BioRad, United States) for confirmation of amplification and determination of size (in terms of bp) of amplified PCR products. The bands were clearly visualized under UV light and the same was analysed on a Gel Documentation System (GelDoc, BioRad, United States). Purification and sequencing of PCR products for all the three genes (*IL-4*, *IL-5*, and *IL-13*) using specific primers were done by outsourcing (Barcode Bio Science, India). After getting the respective sequence, a thorough sequence analysis was done using Laser gene DNAStar software and megablast of Basic local alignment search tool (BLAST, NCBI).

### 2.9. Statistical Analysis

PROC MIXED procedures of SAS [33] were used for analysis of the data and the statistical model consisted of Day (DPI), Group (resistant and susceptible Garole sheep), and Group × Day interactions as main effects with repeated measures of period and animal as a random effect. The following statistical model was used:

Y*i* *j* *k* = *μ* + G*i* + Pj + (G × P)*i* *j* + a*k* + e*i* *j* *k*.

where Y*i* *j* *k* = dependent variable, *μ* = overall mean, G*i* = effect of group *i*, P*j* = effect of period *j*, (G × P)*i* *j* = interaction effect of group *i* and period *j*, a*k* = random effect of animal *k*, and e*i* *j* *k* = overall residual error. Different covariance matrix was tested and the covariance matrix that showed a better model fit was used. When an interaction effect was significant, the ‘SLICE’ option in the SAS model was used to find out the significant (*p* < 0.05) difference among groups in a period or among periods within a group. Subsequently, significant differences (*p* < 0.05) among the groups within a period or among the periods within a group were detected using pair‐wise comparisons specifying ‘PDIFF’ in the least square means (‘LSMEANS’).

Data recorded FEC of experimental sheep were log transformed to reduce right‐skewness and to stabilize variance and the log transformed FEC values assume normally distributed data. Pearson correlation analysis between the FEC and other parameters was done by SPSS (Version 20.0).

## 3. Results

### 3.1. Experimental Haemonchosis, Fecal Egg Count, and Body Weight

**Figure 1:**
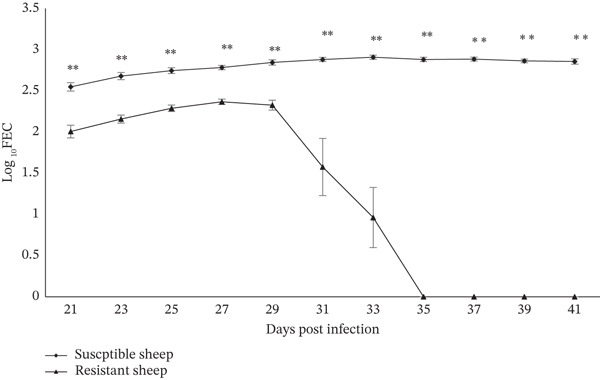
Changes in Faecal egg count (log_10_ FEC) between resistant and susceptible Garole sheep experimentally infected with *Haemonchus contortus*. Note: ∗∗ Indicates a significant difference in log_10_ FEC at *p* < 0.01.

Experimental haemonchosis was developed after challenge infection as described earlier. In the infected resistant sheep, prepatent period ranged from 18 to 21 days with a mean prepatent period of 19.875 (±0.441) days, whereas in infected susceptible sheep, egg of *H. contortus* first appeared between 16 and 19 days and mean prepatent period was found to be 17.625 (±0.375) days. No significant (*p* > 0.05) difference was recorded in prepatent period of infection between the resistant and susceptible sheep. A significantly (*p* < 0.05) lower FEC was recorded in resistant sheep as compared with the susceptible sheep during the entire study period of experimental *H*. contortus infection (Figure 1). Two infected resistant sheep were found to be free of *H. contortus* infection on 31 DPI; another two sheep on 33 DPI and remaining four sheep on 35 DPI as confirmed by absence of *H*. *contortus* eggs during examination of fecal sample. None of the resistant sheep revealed any egg of *H. contortus* during fecal examination from 35 DPI till the end of the study indicating possible clearance of infection (Figure 1). On the other hand, all the infected susceptible sheep exhibited higher FEC till 41 DPI (Figure 1). Sheep of control group did not reveal any eggs of *H*. *contortus* on fecal examination during entire period of experimental infection.

Two‐way ANOVA with repeated measures revealed that group of sheep lacked any significant (*p* > 0.05) effect on body weight of experimental sheep, whereas Day (period of infection) and the interaction between the Day and Group showed significant effect (*p* < 0.001) on body weight (Table 2). In resistant sheep, no significant (*p* > 0.05) changes in body weight occurred due to experimental *H*. *contortus* infection during the entire study period. Consequently, no significant (*p* > 0.05) difference in the body weight was found when compared between the resistant and control sheep as a result of experimental *H. contortus* infection. However, a significant (*p* < 0.05) reduction of the body weight of susceptible sheep was discernible from 20 DPI compared with their preinfection (0 DPI) value and also compared with the control as well as the resistant sheep from 30 DPI to 40 DPI (Table 2).

**Table 2:** Changes in body weight (kg) and haematological parameters in resistant and susceptible Garole sheep experimentally infected with *Haemonchus contortus.*

Variable	Day	Group			SEM	*p*		
		Control sheep	Infected resistant	Infected susceptible		Group	Day	*G* *r* *o* *u* *p* × *D* *a* *y*
Body weight (kg)	0	12.0	11.9	12.0^a^				
	10	12.1	11.9	11.9^a^	0.34	0.16	< 0.001	< 0.001
	20	12.2	11.8	11.2^b^				
	30	12.4^x^	11.9^x^	10.9^cy^				
	40	12.4^x^	11.8^x^	10.5^dy^				
Haemoglobin (gm/dl)	0	12.0	12.0^a^	11.9^a^	0.29	< 0.001	< 0.001	< 0.001
	10	11.9^x^	11.6^abx^	11.0^by^				
	20	11.9^x^	11.4^cx^	10.0^cy^				
	30	11.9^x^	11.5^bcx^	9.55^dy^				
	40	12.1^x^	11.8^abx^	9.20^dy^				
PCV (%)	0	34.1	34.1	34.2^a^	0.26	< 0.001	< 0.001	< 0.001
	10	34.1^x^	33.8^x^	32.9^by^				
	20	33.9^x^	33.4^x^	30.8^cy^				
	30	33.9^x^	33.7^x^	29.3^dy^				
	40	33.9^x^	33.7^x^	29.0^dy^				
PEC (cells/mL)	0	885	863^c^	875^c^	4.54	0.034	< 0.001	< 0.001
	10	953^y^	1401^bx^	1211^bx^				
	20	953^z^	2040^ax^	1626^ay^				
	30	957^z^	1828^ax^	1506^ay^				
	40	930	1199^b^	1228^b^				

*Note:* Mean values bearing superscript letters x, y, and z in a row and a, b, c, d, and e in a column within a variable differ significantly (*p* < 0.05). SEM, pooled standard error mean for the group and day interaction.

Abbreviations: PCV, packed cell volume and PEC, peripheral eosinophil count.

### 3.2. Hematological Values

All the three factors like group of experimental sheep, DPI, and the two‐way interaction between the group and day showed significant (*p* < 0.001) influence on Hb, PCV%, and PEC values (Table 2) in *H. contortus* infected Garole sheep. The PCV values did not differ significantly (*p* > 0.05) in resistant sheep during the period of experimental infection. Although Hb value decreased significantly (*p* < 0.05) in resistant sheep only on 20 DPI compared with their preinfection value (Table 2). Expectedly, Hb, and PCV values did not vary significantly (*p* > 0.05) in control sheep during entire study period. However, significant (*p* < 0.05) reduction of Hb and PCV level of susceptible sheep was observed from 10 DPI compared with infected resistant and control sheep and also compared with their preinfection value (Table 2). A significant (*p* < 0.01) increase in PEC of both resistant and susceptible sheep was recorded from 10 DPI compared with the values recorded during the preinfection period. The control sheep of the present experiment yielded analogous result in PEC value without any significant difference like that of Hb and PCV values. Peripheral eosinophilia was found to be significantly (*p* < 0.01) higher in resistant sheep only on two occasions, that is, 20 and 30 DPI compared with infected susceptible sheep (Table 2).

### 3.3. Serum Th‐2 Cytokine Concentration and Associated Gene Expression

Group of sheep (resistant/susceptible), day (period of infection), and two‐way interaction between the group and day showed significant (*p* < 0.001) effect on Th‐2 cytokine concentration and also on relative expression of associated genes in experimental sheep on different post infection days. Concentration of Th‐2 cytokines (IL‐4, IL‐5, and IL‐13) and mRNA expression of all three Th‐2 cytokine genes increased in both the infected resistant and susceptible sheep after challenged infection with *H. contortus* compared with the control sheep. Relative gene expression of synthesized cDNA was normalized by amplifying *GAPDH* gene.

**Figure 2:**
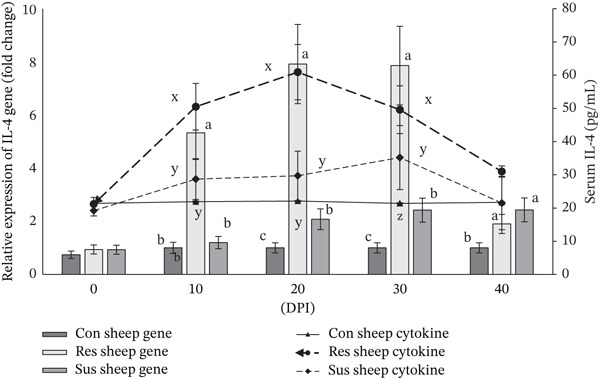
Changes in serum concentration of cytokine IL‐4 and relative expression of *IL-4* gene in resistant and susceptible Garole sheep infected with *Haemonchus contortus*. Note: Letters x, y, z vary significantly (*p* < 0.05) in cytokine concentration and the letters a, b, c vary significantly (*p* < 0.05) in relative expression of cytokine gene among the experimental groups of sheep. Con sheep Gene: gene expression of control sheep; Res sheep Gene: gene expression of infected resistant sheep; Sus sheep Gene: gene expression of infected susceptible sheep; Con sheep cytokine: cytokine concentration of control sheep; Res sheep cytokine: cytokine concentration of infected resistant sheep; and Sus sheep cytokine: cytokine concentration of infected susceptible sheep.

**Figure 3:**
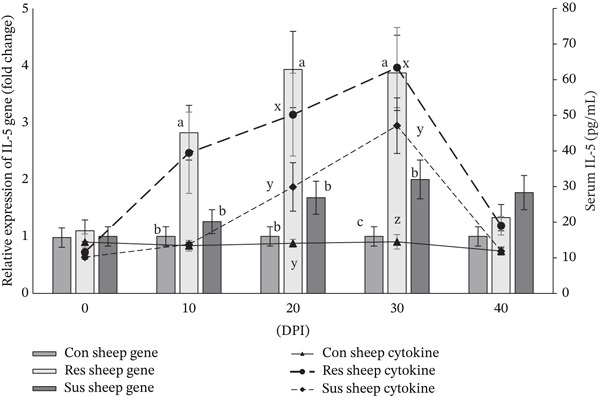
Variation in serum concentration of cytokine IL‐5 and relative expression of *IL-5* gene in *Haemonchus contortus* infected resistant and susceptible Garole sheep on different post infection days. Note: Letters x, y, z vary significantly (*p* < 0.05) in cytokine concentration and the letters a, b, c vary significantly (*p* < 0.05) in relative expression of cytokine gene among the experimental groups of sheep. Con sheep Gene: gene expression of control sheep; Res sheep Gene: gene expression of infected resistant sheep; Sus sheep Gene: gene expression of infected susceptible sheep; Con sheep cytokine: cytokine concentration of control sheep; Res sheep cytokine: cytokine concentration of infected resistant sheep; and Sus sheep cytokine: cytokine concentration of infected susceptible sheep.

**Figure 4:**
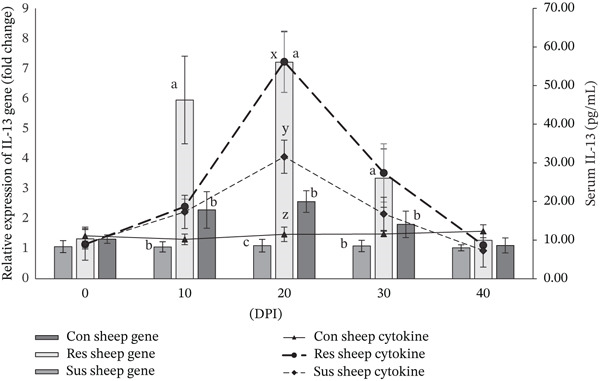
Comparison of serum cytokine IL‐13 concentration and relative expression of *IL-13* gene between resistant and susceptible Garole sheep infected with *Haemonchus contortus*. Note: Letters x, y, z vary significantly (*p* < 0.05) in cytokine concentration and the letters a, b, c vary significantly (*p* < 0.05) in relative expression of cytokine gene among the experimental groups of sheep. Con sheep Gene: gene expression of control sheep; Res sheep Gene: gene expression of infected resistant sheep; Sus sheep Gene: gene expression of infected susceptible sheep; Con sheep cytokine: cytokine concentration of control sheep; Res sheep cytokine: cytokine concentration of infected resistant sheep; and Sus sheep cytokine: cytokine concentration of infected susceptible sheep.

Serum concentration of cytokine IL‐4 increased significantly (*p* < 0.01) in the resistant sheep from 10 DPI onwards to 30 DPI whereas compared with the preinfection value and also with the values recorded for susceptible as well as control sheep (Figure 2). Interestingly, the relative expression of *IL-4* gene in peripheral blood of resistant sheep was also significantly (*p* < 0.01) higher compared with susceptible sheep from 10 to 30 days after the challenge infection (Figure 2). A higher value of IL‐5 cytokine concentration (*p* < 0.01) was documented in infected resistant sheep from 20 to 30 DPI compared with susceptible sheep infected with *H*. *contortus* (Figure 3). Although significant (*p* < 0.01) upregulation of *IL-5* gene was observed in resistant sheep from 10 to 30 DPI compared with susceptible sheep due to induced haemonchosis (Figure 3). Resistant Garole sheep showed increased (*p* < 0.01) concentration of IL‐13 cytokine only on 20 DPI compared with susceptible sheep with induced haemonchosis (Figure 4). However, elevated level of *IL-13* gene expression (*p* < 0.01) was also observed like other two genes (*IL-4* and *IL-5* gene) in peripheral blood of resistant sheep compared with susceptible Garole sheep from 10 to 30 DPI (Figure 4). In both the resistant and susceptible sheep, cytokine (IL‐4, IL‐5, and IL‐13) levels in serum were positively (*p* < 0.01) correlated with the expressions of the related genes (*IL-4*, *IL-5*, and *IL-13*). Concentrations of all the cytokines and relative expressions of all three cytokine genes were significantly (*p* < 0.01) and positively correlated with the PEC value in resistant sheep (Table 3). Although in susceptible sheep, cytokine IL‐5 was positively correlated (*p* < 0.01) with Hb and PEC and cytokine IL‐13 was recorded to be correlated with the PCV in our study (Table 4).

**Table 3:** Correlation of FEC with other parameters in resistant Garole sheep experimentally infected with *Haemonchus contortus.*

		FEC	Hb	PCV	Bwt	PEC	cIL4	cIL5	cIL13	gIL4	gIL5	gIL13
FEC	Pearson correlation	1										
	Sig. (2‐tailed)											
Hb	Pearson correlation	—0.411 ^∗^	1									
	Sig. (2‐tailed)	0.046										
PCV	Pearson correlation	−0.370	0.661 ^∗∗^	1								
	Sig. (2‐tailed)	0.075	0.000									
Bwt	Pearson correlation	0.076	0.627 ^∗∗^	0.202	1							
	Sig. (2‐tailed)	0.723	0.001	0.345								
PEC	Pearson correlation	0.670 ^∗∗^	−0.347	−0.035	−0.220	1						
	Sig. (2‐tailed)	0.000	0.096	0.870	0.302							
cIL4	Pearson correlation	0.459 ^∗^	−0.323	0.050	−0.154	0.486 ^∗^	1					
	Sig. (2‐tailed)	0.024	0.124	0.818	0.473	0.016						
cIL5	Pearson correlation	0.354	−0.133	−0.025	−0.036	0.435 ^∗^	0.377	1				
	Sig. (2‐tailed)	0.089	0.537	0.906	0.866	0.034	0.070					
cIL13	Pearson correlation	0.714 ^∗∗^	−0.258	−0.239	0.086	0.651 ^∗∗^	0.498 ^∗^	0.476 ^∗^	1			
	Sig. (2‐tailed)	0.000	0.224	0.260	0.691	0.001	0.013	0.019				
gIL4	Pearson correlation	0.568 ^∗∗^	−0.014	−0.020	0.116	0.512 ^∗^	0.537 ^∗∗^	0.736 ^∗∗^	0.582 ^∗∗^	1		
	Sig. (2‐tailed)	0.004	0.947	0.927	0.590	0.010	0.007	0.000	0.003			
gIL5	Pearson correlation	0.204	0.046	0.193	−0.091	0.558 ^∗∗^	0.301	0.605 ^∗∗^	0.417 ^∗^	0.552 ^∗∗^	1	
	Sig. (2‐tailed)	0.340	0.832	0.365	0.672	0.005	0.153	0.002	0.043	0.005		
gIL13	Pearson correlation	0.641 ^∗∗^	−0.102	−0.059	0.182	0.552 ^∗∗^	0.545 ^∗∗^	0.407 ^∗^	0.937 ^∗∗^	0.591 ^∗∗^	0.395	1
	Sig. (2‐tailed)	0.001	0.634	0.786	0.394	0.005	0.006	0.048	0.000	0.002	0.056	

Abbreviations: Bwt, body weight; cIL4, cytokine IL‐4 concentration; cIL5, cytokine IL‐5 concentration; cIL13, cytokine IL‐13 concentration; FEC, fecal egg count; gIL4, relative expression of cytokine *IL-4* gene; gIL5, relative expression of cytokine *IL-5* gene; gIL13, relative expression of cytokine *IL-13* gene Hb, hemoglobin; PCV, packed cell volume; and PEC, peripheral eosinophil count.

∗∗Correlation is significant at the 0.01 level (2‐tailed).

∗Correlation is significant at the 0.05 level (2‐tailed).

**Table 4:** Correlation of FEC with other parameters in susceptible Garole sheep experimentally infected with *Haemonchus contortus.*

		FEC	Hb	PCV	Bwt	PEC	cIL4	cIL5	cIL13	gIL4	gIL5	gIL13
FEC	Pearson Correlation	1										
	Sig. (2‐tailed)											
Hb	Pearson Correlation	−0.367	1									
	Sig. (2‐tailed)	0.078										
PCV	Pearson Correlation	−0.525 ^∗∗^	0.934 ^∗∗^	1								
	Sig. (2‐tailed)	0.008	0.000									
Bwt	Pearson Correlation	−0.246	0.610 ^∗∗^	0.642 ^∗∗^	1							
	Sig. (2‐tailed)	0.246	0.002	0.001								
PEC	Pearson Correlation	−0.289	0.748 ^∗∗^	0.726 ^∗∗^	0.721 ^∗∗^	1						
	Sig. (2‐tailed)	0.172	0.000	0.000	0.000							
cIL4	Pearson Correlation	0.015	0.028	−0.027	0.254	−0.020	1					
	Sig. (2‐tailed)	0.945	0.896	0.900	0.231	0.925						
cIL5	Pearson Correlation	−0.030	0.501 ^∗^	0.293	0.354	0.537 ^∗∗^	0.342	1				
	Sig. (2‐tailed)	0.890	0.013	0.164	0.090	0.007	0.102					
cIL13	Pearson Correlation	−0.209	0.366	0.443 ^∗^	0.168	0.163	0.053	−0.049	1			
	Sig. (2‐tailed)	0.327	0.079	0.030	0.432	0.446	0.806	0.819				
gIL4	Pearson Correlation	0.105	−0.177	−0.187	0.158	−0.137	0.807 ^∗∗^	−0.013	−0.115	1		
	Sig. (2‐tailed)	0.624	0.407	0.380	0.462	0.524	0.000	0.953	0.593			
gIL5	Pearson Correlation	−0.114	0.252	0.086	−0.174	0.359	−0.178	0.453 ^∗^	−0.181	−0.249	1	
	Sig. (2‐tailed)	0.596	0.235	0.689	0.416	0.085	0.406	0.026	0.397	0.240		
gIL13	Pearson Correlation	−0.409 ^∗^	0.188	0.347	0.126	−0.020	0.366	0.027	0.649 ^∗∗^	0.137	−0.286	1
	Sig. (2‐tailed)	0.047	0.378	0.096	0.558	0.924	0.079	0.899	0.001	0.524	0.176	

Abbreviations: Bwt, body weight; cIL4, cytokine IL‐4 concentration; cIL5, cytokine IL‐5 concentration; cIL13, cytokine IL‐13 concentration; FEC, faecal egg count; Hb, haemoglobin; PCV, packed cell volume; gIL4, relative expression of cytokine *IL-4* gene; gIL5, relative expression of cytokine *IL-5* gene; gIL13, relative expression of cytokine *IL-13* gene; and PEC, peripheral eosinophil count.

∗∗Correlation is significant at the 0.01 level (2‐tailed).

∗Correlation is significant at the 0.05 level (2‐tailed).

### 3.4. Sequence Analyses of Th‐2 Cytokine Genes Between Resistant and Susceptible Sheep

The amplicons of *IL-4*, *IL-5* and *IL-13* genes as obtained by conventional PCR were 741 bp (Figure S6), 618 bp (Figure S7), and 524 bp (Figure S8), respectively. All three Th‐2 cytokine genes are located on Chromosome Number 5. The partial sequences of *IL-4*, *IL-5*, and *IL-13* genes underwent multiple sequence alignment with similar reference genes of these three cytokines, that is, ovine *IL-4* gene sequence (NC056058.1), ovine *IL-5* gene sequence (NC056058.1), and ovine *IL-13* gene sequence (NC056058.1), respectively. Multiple sequence alignment study depicted six single nucleotide variations (SNV) at 117 bp, 159 bp, 178 bp, 193 bp, 311 bp, and 437 bp in the *IL-4* gene. Five SNVs were found in the *IL-5* gene at 19 bp, 49 bp, 81 bp, 100 bp, and 154 bp regions, and in the *IL-13* gene two SNVs were recorded at 132 bp and 467 bp regions. These data were revealed as compared to their reference gene sequences. The sequences have been submitted to the NCBI, GenBank and accession numbers for the sequences have been obtained (PV008714 to PV008767). No SNV was observed either only in resistant sheep or in susceptible sheep but was intermingled in both resistant and susceptible sheep. Therefore, the SNVs observed in the three genes (*IL-4*, *IL-5*, and *IL-13*) could demonstrate to be associated with the phenotypic expression of resistance and susceptibility in Garole sheep. This observation needs further study on whole gene sequence analyses of the three cytokine (*IL-4*, *IL-5*, and *IL-13*) genes involving a greater number of resistant and susceptible Garole sheep selected on the basis of FEC.

### 3.5. Correlation of FEC With Other Parameters

Although correlation of FEC with other parameters was done in the present study, a significantly (*p* < 0.05) negative correlation was recorded between FEC and Hb in resistant sheep (Table 3) and between FEC and PCV in susceptible sheep (*p* < 0.010) experimentally infected with *H*. *contortus* (Table 4). In the resistant group, FEC was positively correlated (*p* < 0.01) with PEC, cytokine IL‐4, and IL‐13 concentration in serum and also with the expression level of those two cytokine genes (*IL-4* and *IL-13*) in peripheral blood (Table 3). Whereas in susceptible sheep, FEC was negatively correlated (*p* < 0.05) only with the expression level of *IL-13* gene (Table 4).

## 4. Discussion

The strategic exploitation of host genetic factors conferring resistance to the hosts against GIN infection is one of the most promising approaches for circumventing the escalating challenge of drug resistance. Host immune response is known to play an important role in the host′s resistance against GIN. Therefore, studying host immune response in terms of different immunological components during an infection is the key to determine the mechanisms underlying host resistance against GIN.

Garole sheep of Sundarban delta of West Bengal showed within‐breed resistance to GIN in our preliminary studies [17–23] and the results of those studies encouraged us to find out more logistic and scientific base embedded in this phenomenon. Semi‐intensive system of management practice for Garole sheep in an endemic area like Sundarban delta might be responsible for individual resistance in Garole sheep against GIN by influencing exposure levels and the sheep′s immune response. Resistant and susceptible Garole sheep for the present study were selected on the basis of FEC, the most important phenotypic indicator of resistance, and PCV, and body weight, important indicators of host resilience. Measurement of FEC is an approximate method of determining the intensity of infection as it is related to the fecundity of female worms only [34]. In some other reference works, FEC and PCV were also taken into account [35, 36] for determination of host′s resistance against haemonchosis. Hence, in our study, we have taken these two parameters for the relevant assessment. Analysis of FEC yielded a discernible picture in which resistant sheep revealed significantly (*p* < 0.05) lower FEC than the susceptible sheep during 1 year of field study. Body weight and PCV of all comparatively resistant and susceptible sheep were measured for final selection because these two parameters are evidently considered key phenotypic markers for identification of host resilience. The resistant sheep exhibited persistently low FEC and comparatively higher body weight and PCV than the susceptible sheep [20, 37]. Our present finding related to within‐breed resistance corroborates with the findings of other researchers who worked in other breeds, that is, Australian Merino sheep [38], INRA 401 [39], Scottish Black Face [40] and Rasa Aragonesa [41] and in Garole sheep of different locations [20].

Although no significant (*p* > 0.05) difference was observed in prepatent period between the resistant and susceptible sheep after experimental infection with *H*. *contortus*, FEC was significantly (*p* < 0.01) lower in resistant sheep (Figure 1) compared with susceptible ones [41, 42]. Host′s acquired immune response might be attributed to significant variation in FEC within a breed of sheep in the same age group [43]. Absence of *H*. *contortus* eggs in fecal samples of infected resistant sheep from 31 to 35 DPI indicates elimination or rejection of adult worms from the abomasum of hosts. This observation reconfirms our previous finding of spontaneous elimination of experimental *H. contortus* infection in resistant Garole sheep [19–22]. An appreciable statement pertaining to increased concentration of mast cells and globule leucocytes in abomasal mucosa and participation of parasite‐specific antibodies (IgA, IgG1, and IgE) leading to rejection of challenged larvae of *H*. *contortus* in sheep was asserted by earlier workers [44, 45]. However, mechanisms involved in elimination of adult worms in the present study warrant further detailed investigation encompassing the association of those cells (mast cells and globule leucocytes) and parasite‐specific immunoglobulin with the rejection of *H*. *contortus* from the abomasum of the host.

Body weight is considered to be a useful indicator of host resilience to *H*. *contortus* in several breeds of sheep, as has been established by different workers [46, 47]. No marked difference was observed in body weight of resistant sheep, but significant (*p* < 0.05) attenuation of body weight of susceptible sheep was conspicuously evident, which might be due to a higher rate of *H*. *contortus* infection, as reflected by higher FEC. Significantly (*p* < 0.05) higher body weight in resistant sheep compared with susceptible sheep has been recorded earlier [22, 48, 49]. A negative correlation of body weight and FEC was observed in the resistant breed of sheep [48]. In infected susceptible Garole sheep, a negative correlation between FEC and body weight was observed, but this correlation was not significant (*p* > 0.05) in the present study (Table 4).

Our present finding of comparatively higher value of Hb and PCV in resistant sheep compared with the susceptible sheep (Table 2) substantiates with findings of other workers [35, 49]. PCV has been considered as an indicator of host resilience against *H*. *contortus* and it has shown heritability ranging from 0.15 to 0.36 for *H*. *contortus* resistance [37, 50]. Comparatively higher Hb and PCV in infected resistant sheep apparently indicated their resilience power to tolerate the harmful effects of *H*. *contortus*. FEC was found to be negatively correlated with Hb in resistant sheep (Table 3) and with PCV in susceptible sheep (Table 4), and this finding fairly aligns with the finding of Brahma et al. [20] and Saddiqi et al. [47].

Eosinophils are considered the key effector cells playing a crucial role in GIN infection, and the researchers always ponder on the immunological trajectory influenced by these cells while studying host immunity against GIN. Eosinophils play an important role in resistance to GIN infection through the release of toxic granular proteins and production of IL‐4 and IL‐13 [51] from Th‐2 type cells and thus strengthen the Th‐2 type immune response. In resistant sheep, significant (*p* < 0.05) peripheral eosinophilia had been found to be positively correlated with the cytokine levels and expression of cytokine genes in peripheral blood. Production and activation of eosinophils are mainly induced by the cytokine IL‐5, which was significantly (*p* < 0.05) increased in resistant sheep. The increased number of eosinophil counts in the peripheral blood of resistant sheep had been associated with low establishment of *H*. *contortus* or its elimination [13] and low FEC and worm burden in resistant sheep [22]. The period of peripheral eosinophilia coincided with the spontaneous elimination of parasites, as confirmed by fecal sample examination in resistant sheep. Therefore, it could be suggested that eosinophils, along with Th‐2 cytokines (IL‐4, IL‐5, and IL‐13), might be associated with the resistance of Garole sheep against *H*. *contortus*.

Although it is believed that the host immune system contributes to the manifestation of inherited resistance, the actual mechanism of the host′s immune reaction is still not well understood. Many authors suggested that Th‐2 type immune responses are associated with GIN resistance in sheep [52, 53] and it works through the release of different cytokines and production of specific antibodies [6]. Th‐2 type immune response characterized by increased production of eosinophils, mast cells, antibodies as well as increased production of IL‐4, IL‐5, and IL‐13 has been reported [53] in resistant sheep infected with GIN including *H*. *contortus* [54].

The concentration of all the three Th‐2 cytokines (IL‐4, IL‐5, and IL‐13) increased significantly (*p* < 0.01) in the resistant sheep compared with the susceptible sheep at different time points of induced haemonchosis (Figures 2, 3, and 4, respectively). This finding expectedly exhibits congruence with the increased relative expression of the target genes (*IL-4*, *IL-5,* and *IL-13*) in resistant sheep than the susceptible Garole sheep in our present experiment. Significant upregulation of relative expression of all the three cytokine genes might have resulted in increased gene products, that is, the cytokine (IL‐4, IL‐5, an IL‐13) proteins in resistant sheep. Existing immunological knowledge states that the cytokine IL‐4 promotes the development of Th‐2 type cells which release other cytokines including IL‐5 and IL‐13. also stimulates the B cells to proliferate and differentiate to produce antibody particularly IgE. Proliferation and activation of eosinophils are stimulated by cytokine IL‐5, which stimulates the production of IgA [8]. On other hand, IL‐13 plays an important role in mucous production and regeneration of intestinal epithelium during GIN infection [55]. Increased mucous play a primary role in the physical expulsion of the worms from the GI tract because of its thick and viscid nature as observed in our study.

A couple of works were carried out to account cytokine gene expression [20–22] in host resistance against GIN infection [56–61] in livestock. The underlying findings of those experiments share similarities and the core proposition of these findings supports the basic understandings, but still there have been many controversies over cytokine expression profiles in the immune response against GIN. This is due to the lack of a standard immune response profile against each parasite species. Pernthaner et al. [61] recorded higher expression level of *IL-5* and *IL-13* genes in resistant sheep, but they did not observe any change in expression of the *IL-4* gene. Whereas Shakya et al. [58] reported marked expression of the *IL-4* gene and Escribano et al. [59] observed significant upregulation of *IL-4, IL-5* and *IL-13* genes in PBMC of resistant sheep compared with susceptible sheep. Elevated expression of *IL-4* and *IL-5* genes in abomasal mucosa, tissue, and lymph nodes have been observed in resistant sheep but those genes were not expressed in susceptible sheep [61]. In our present study, higher Th‐2 type immune response in terms of increased peripheral eosinophilia and serum concentration of Th‐2 cytokines (IL‐4, IL‐5, and IL‐13) and upregulation of Th‐2 cytokine (*IL-4*, *IL-5*, and *IL-13*) gene expression in PBMC of resistant Garole sheep is conspicuously evident. Although no general agreement on polarization of host immune response against GIN has been achieved, it is widely accepted that resistance to GIN varies between the breeds of animals [62, 63] and also between parasites [64]. Therefore, more and more research works should be carried out to reinforce the fundamental insights about the development of host′s resistance against *H*. *contortus*. Our team is consistently working in this direction and some major aspects have already been unfurled as mentioned earlier. In the earlier works, we have already reported the association of cytokines IFN‐*γ* and IL‐10 concentration in serum and relative expression of *IFN-γ* and *IL-10* genes with *H*. *contortus* resistance in Garole sheep [21]. The findings of the present study further elaborate our previous findings on host resistance in Garole sheep.

Although the Th‐2 cytokine concentration in serum and associated Th‐2 cytokine gene expression in PBMC varied significantly between the resistant and susceptible sheep, no marked difference was observed in the partial sequence of any of the three Th‐2 cytokine (*IL-4*, *IL-5*, and *IL-13*) genes in the present study. SNVs were observed in all the three genes, but none of these nucleotide variations of any gene could be suggested to be associated with resistance or susceptibility to *H*. *contortus* infection in Garole sheep. Upregulation of Th‐2 cytokine genes resulted in increased production of Th‐2 cytokines, particularly IL‐4, which promotes Th‐2 type immune response against *H. contortus* infection in resistant sheep by stimulating proliferation of different effector cells, production of antibodies, and proliferation of intestinal goblet cells producing increased mucous stimulated by the cytokine IL‐13. All these effects might have created an adverse condition locally [6, 56] leading to elimination of the parasite.

## 5. Conclusion

The present study demonstrated a clear association of peripheral or systemic Th‐2 type immune response with the host resistance against *H*. *contortus* in Garole sheep. Spontaneous elimination of *H. contortus* from resistant Garole sheep was contemporaneous with the increased concentration of Th‐2 cytokine proteins and relative expression of Th‐2 cytokine genes and eosinophilia in peripheral circulation. These findings might contribute to the growing understanding of the complex interplay between the host immune response and resistance against GIN. However, observation of our present study warrants further in situ investigation to study the local immune response in the abomasum of resistant sheep infected with *H. contortus*.

## Author Contributions

Ananta Hembram: methodology and data curation; Supradip Das: investigation and data curation; Soumitra Pandit: data curation; Surajit Baidya: resource; Abhijit Nandi: formal analysis and data curation; Subhas Chandra Mandal: writing—review and editing; Shyam Sundar Kesh: methodology and investigation; Shamik Polley: visualization; Amlan Patra: data analysis and formal analysis; Ayan Mukherjee: data curation; and Ruma Jas: conceptualization, project administration, funding acquisition, and writing—original draft.

## Funding

This work was supported by WB University of Animal & Fishery Sciences.

## Disclosure

All the authors read and approved the manuscript.

## Ethics Statement

The animals used in the present study were strictly maintained at par with standard care guideline established by the Institutional Animal Ethics committee (IAEC) of West Bengal University of Animal and Fishery Sciences (Regd No. 763/GO/Re/SL/03/CCSEA). The present project proposal (No. 763/GO/Re/SL/03/35/2022‐23) was duly approved by the IAEC, Faculty of Veterinary and Animal Sciences, West Bengal University of Animal and Fishery Sciences, Kolkata 700037.

## Conflicts of Interest

The authors declare no conflicts of interest.

## Supporting information


**Supporting Information** Additional supporting information can be found online in the Supporting Information section. Additional supporting information can be obtained online under the Supporting Information section. Figure S1. Agarose gel (1%) electrophoresis of total RNA extracted from peripheral blood lymphocytes of experimental Garole sheep. Figure S2. Agarose gel (1%) electrophoresis of RT‐PCR product of *GAPDH* gene of different experimental groups Garole sheep on different postinfection days. Figure S3. Agarose gel (1%) electrophoresis of RT‐PCR product of *IL-4 g*ene of different groups of experimental Garole sheep. Figure S4. Agarose gel (1%) electrophoresis of RT‐PCR product of *IL–5* gene of different experimental groups Garole sheep. Figure S5. Agarose gel (1%) electrophoresis of RT‐PCR product of *IL–13* gene of different experimental groups Garole sheep. Figure S6. Agarose gel (1%) electrophoresis of partially amplified *IL-4* gene (741 bp) of Garole sheep obtained by conventional PCR. Figure S7. Agarose gel (1%) electrophoresis of partially amplified *IL-5* gene (618 bp) of Garole sheep obtained by conventional PCR. Figure S8. Agarose gel (1%) electrophoresis of partially amplified *IL-13* gene (524 bp) of Garole sheep obtained by conventional PCR.

## Data Availability

The data generated in this study are available from the corresponding author on a reasonable request.
