# Supplementary material for: Comparison of Th‐2 Cytokines and Associated Gene Expression in Peripheral Blood of Resistant and Susceptible Garole Sheep Infected With Haemonchus contortus
Source: J Parasitol Res. 2026 Apr 15;2026:3052032. doi: 10.1155/japr/3052032 (PMC13080255; doi:10.1155/japr/3052032)
Supplement: Supplementary file 1 — Supporting Information Additional supporting information can be found online in the Supporting Information section. Additional supporting information can be obtained online under the Supporting Information section. Figure S1. Agarose gel (1%) electrophoresis of total RNA extracted from peripheral blood lymphocytes of experimental Garole sheep. Figure S2. Agarose gel (1%) electrophoresis of RT‐PCR product of GAPDH gene of different experimental groups Garole sheep on different postinfection days. Figure S3. Agarose gel (1%) electrophoresis of RT‐PCR product of IL-4 gene of different groups of experimental Garole sheep. Figure S4. Agarose gel (1%) electrophoresis of RT‐PCR product of IL–5 gene of different experimental groups Garole sheep. Figure S5. Agarose gel (1%) electrophoresis of RT‐PCR product of IL–13 gene of different experimental groups Garole sheep. Figure S6. Agarose gel (1%) electrophoresis of partially amplified IL-4 gene (741 bp) of Garole sheep obtained by conventional PCR. Figure S7. Agarose gel (1%) electrophoresis of partially amplified IL-5 gene (618 bp) of Garole sheep obtained by conventional PCR. Figure S8. Agarose gel (1%) electrophoresis of partially amplified IL-13 gene (524 bp) of Garole sheep obtained by conventional PCR. [file JAPR-2026-3052032-s001.docx]

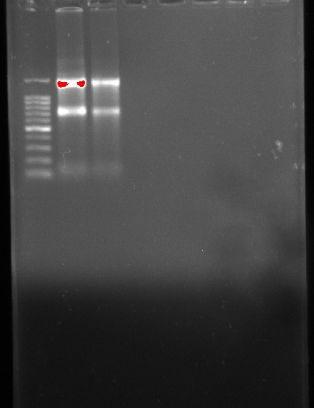


28 S

18 S

5 S

Figure S1: Agarose gel (1%) electrophoresis of total RNA extracted from peripheral blood lymphocytes of experimental Garole sheep


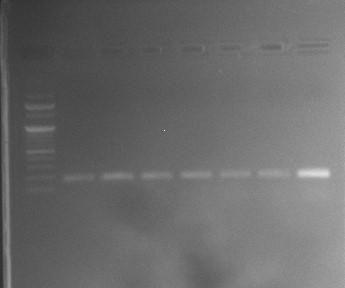


100 bp ladder

176 bp

Figure S2: Agarose gel (1%) electrophoresis of RT-PCR product of GAPDH gene of different experimental groups Garole sheep on different post infection days

100 bp ladder

190 bp

Figure S3: Agarose gel (1%) electrophoresis of RT-PCR product of IL- 4 *g*ene of different groups of experimental Garole sheep


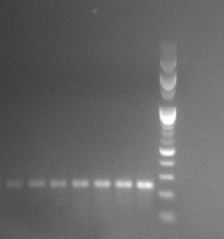


50 bp ladder

137 bp

Figure S4: Agarose gel (1%) electrophoresis of RT-PCR product of IL – 5 gene of different experimental groups Garole sheep


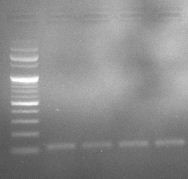


50 bp ladder

51 bp

Figure S5: Agarose gel (1%) electrophoresis of RT-PCR product of IL – 13 gene of different experimental groups Garole sheep


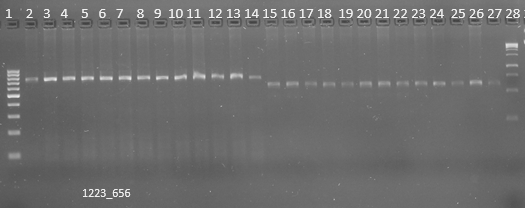


741bp

Figure S6: Agarose gel (1%) electrophoresis of partially amplified IL-4 (741 bp) of Garole sheep obtained by conventional PCR

**
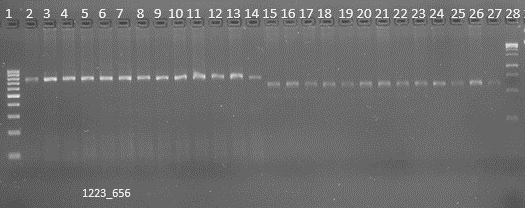
**

618 bp

Figure S7: Agarose gel (1%) electrophoresis of partially amplified IL-5 (618 bp) of Garole sheep obtained by conventional PCR

**
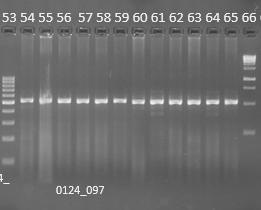
**

524 bp

Figure S8: Agarose gel (1%) electrophoresis of partially amplified IL-13 (524 bp) of Garole sheep obtained by conventional PCR
